# Repurposing the PDMA-approved drugs in Japan using an insect model of staphylococcal infection

**DOI:** 10.1093/femsmc/xtac014

**Published:** 2022-04-26

**Authors:** Atsushi Miyashita, Shuhei Mitsutomi, Tohru Mizushima, Kazuhisa Sekimizu

**Affiliations:** Institute of Medical Mycology, Teikyo University, 359 Otsuka, Hachioji, Tokyo 191-0359, Japan; Institute of Medical Mycology, Teikyo University, 359 Otsuka, Hachioji, Tokyo 191-0359, Japan; LTT Biopharma Co., Ltd., 1-2-20 Kaigan, Minato, Tokyo 105-0022, Japan; School of Pharma-Science, Teikyo University, 359 Otsuka, Hachioji, Tokyo 191-0359, Japan; Genome Pharmaceuticals Institute Co., Ltd., 1-27-8 Hongo, Tokyo 113-0033, Japan

**Keywords:** drug discovery, invertebrate model, drug repositioning, silkworm, Staphylococci

## Abstract

A total of 1253 compounds approved as therapeutic drugs in Japan (Pharmaceuticals and Medical Devices Agency (PMDA)-approved compounds) were screened for their therapeutic effects against *Staphylococcus aureus* infection using the silkworm infection model. In the first stage of screening with an index of prolonged survival, 80 compounds were identified as hits. Of these, 64 compounds were clinically used as antimicrobial agents, and the remaining 16 compounds were not. The 16 compounds were examined for their dose-dependent therapeutic effects on the silkworm model as a second screening step, and we obtained five compounds as a result. One of the compounds (capecitabine) had no documented *in vitro* minimum inhibitory concentration (MIC) value against *S. aureus*. The MIC value of capecitabine against *S. aureus* strains ranged from 125 to 250 µg/ml, and capecitabine was therapeutically effective at a dose of 200 mg/kg in a murine model of *S. aureus* infection. These results suggest that silkworm-based drug repositioning studies are of potential value. Furthermore, the therapeutic effects of capecitabine demonstrated in this study provide an important scientific rationale for clinical observational studies examining the association between staphylococcal infection events and capecitabine administration in cancer chemotherapy patients.

## Introduction

The search for novel pharmaceutical treatments is important in fulfilling unmet medical needs. The development of new therapeutics usually requires exploratory preclinical studies, followed by safety studies and multiple clinical trials to validate the therapeutic effect in humans, resulting in demandingly substantial research and development (R&D) costs (Miyashita and Sekimizu 2021, Miyashita et al. 2021a). However, if a compound has already been approved and used in humans, the cost of clinical development can be significantly reduced because studies on safety and pharmacokinetics in humans for the compound had already been completed. This type of approach to drug discovery is called drug repurposing or drug repositioning (Ashburn and Thor 2004, Kikuchi et al. 2015, Sugizaki et al. 2019).

In drug repositioning, a library of compounds that have already been approved as therapeutic agents is screened for application to new diseases. *In vitro* studies or murine experiments are often used as screening platforms, but *in vitro* studies have the problem of not being able to assess organismal-level responses such as pharmacodynamics and pharmacokinetics. *In vivo* experiments using murine animal models are costly and ethically problematic when conducting large-scale exploratory experiments. We propose that these problems can be overcome by using the silkworm (*Bombyx mori*) models (Kaito et al. 2002, 2005, Orihara et al. 2008, Matsumoto et al. 2011, Miyashita et al. 2012, 2014, 2015, Miyashita and Sekimizu 2021). For silkworms, quantitative sample injection is relatively easy, and the pharmacological effects of compounds can be evaluated at the individual level as demonstrated in the previous study (Hamamoto et al. 2015, Usui et al. 2016). Furthermore, the silkworm-based experimental platform has the advantage that the cost of the experiment is extremely small compared to those using murine models. In addition, with the average size of a laboratory at universities and national research institutes, it is possible to examine up to 100 compounds per day for compound screening using silkworms (*n* = 3/compound, using 300 silkworms). The size of the library used for drug repositioning is usually several hundreds to about a thousand compounds, and when the silkworm is used as a screening platform, it becomes a research project that takes several months to complete, including secondary screening and concurrent efforts with other projects. This feature of the silkworm-based research platform potentially makes it accessible to research groups with limited resources.

In this study, we examined whether there are compounds with therapeutic effects against *Staphylococcus aureus* among compounds in the library of approved drugs that are not classified as antibacterial agents, in order to assess the validity of drug repositioning studies using silkworm models. Here, we found five anticancer compounds with therapeutic activity against staphylococcal infection. Among them, capecitabine, whose minimum inhibitory concentration (MIC) value against *S. aureus* had not been described in the literature, was tested for its therapeutic effect using a murine model.

## Materials and methods

### Silkworms

Silkworms (*B. mori*) were reared as previously described (Miyashita et al. 2012, 2014, 2015, 2021b, Kaito et al. 2020). Fertilized eggs of silkworm (Fu-Yo × Tsukuba-Ne) were purchased from Ehime Sanshu (Ehime, Japan). The hatched larvae were reared at 27°C and fed with Silkmate 2S artificial diet for silkworms (Nihon Nosan Kogyo, Tokyo, Japan) until the fourth instar. After transitioning to the fifth instar stage, they were fed Chisan Nirei artificial diet for silkworms (Katakura Kogyo, Tokyo, Japan).

### Bacterial strains and the Pharmaceuticals and Medical Devices Agency (PMDA)-approved compound library

In this study, methicillin-susceptible *S. aureus* strain MSSA1 (Paul Dzoyem et al. 2013), methicillin-resistant *S. aureus* strains MRSA3, MRSA4, MRSA5, MRSA6, MRSA8, MRSA9, MRSA11, and MRSA12 (Paul Dzoyem et al. 2013), and the Smith strain (Hamamoto et al. 2015), RN4220 (Kaito et al. 2002), Newman (Kaito and Sekimizu 2007), and USA300 (Kaito et al. 2011) were used. A library of Pharmaceuticals and Medical Devices Agency (PMDA)-approved drugs containing 1253 compounds was provided by LTT Bio-Pharma Co., Ltd. (Tokyo, Japan). This study was conducted in a double-blind manner and neither the project manager nor the experimenter was informed of the compound names. For the hit compounds obtained from the initial screening, the compound names were disclosed by the library providers for further study. Therefore, the full list of 1253 compounds was not disclosed by the library provider, but the 80 hit compounds obtained in the initial screening are shown in Table S1 (Supporting Information).

### Screening using silkworm infection model

In the first screening, silkworms injected with 1 × 10^8^ CFU/larva of MSSA1 strain were injected with 0.01 µmol/larva of the compound (*n* = 3/compound). The silkworms were then reared at 27°C and monitored for their survival for 2 days. Compounds that lead to greater survival at 24 hours compared to the vehicle-injected group were considered as hit compounds in the first screening. In the first screening, this experiment was repeated twice, and the compounds that showed reproducible therapeutic effects between the two replicates were counted as first hits. We obtained 80 hits from the first screening and chose 16 nonantibiotic compounds for the second screening. In the second screening, different doses (0, 0.01, 0.1, or 0.5 µmol/larva) were administered to the silkworm (*n* = 3/dose), and injected with 1 × 10^8 ^CFU/larva of MSSA1 strain. The silkworms were then reared at 27°C for 2 days and monitored for survival.

### Measurement of antimicrobial activity (MIC value)

In our study, MIC values were determined by microdilution method using Mueller–Hinton Broth (Hamamoto et al. 2015). Briefly, an aliquot of 100 μl of Mueller–Hinton Broth containing live *S. aureus* (10^3^-fold dilution of overnight culture) was placed in the wells of a 96-well round-bottom disposable plate. Then, 100 μl/well of a 2-fold serial dilution of capecitabine was added, mixed, and incubated at 37°C overnight. The MIC was defined as the lowest concentration that inhibits the growth of *S. aureus*. In this experiment, Vancomycin was used as the positive control, and the MIC value of vancomycin was confirmed to be 0.8–3.1 µg/ml in each trial.

### Evaluation of the therapeutic effect of capecitabine in mice

To a 4-week-old female ICR mice (Oriental Yeast Co., Ltd., Tokyo, Japan), 0.4 ml Smith live cells (an overnight culture containing 4 × 10^7^ CFU) was intraperitoneally administered, and immediately afterwards capecitabine dissolved in MilliQ water was intraperitoneally administered at a dose of 0, 200, or 500 mg/kg (volume = 0.4 ml/mouse). A total of six animals were used for the no-drug control group, and seven animals were used for the two treatment groups (200 and 500 mg/kg capecitabine groups). To minimize the sacrifice of mice, we did not run a group treated with capecitabine alone (i.e. without infection) to assess its toxicity. The Log-rank test was performed for the difference in survival time between groups. The statistical analysis was performed using the package ’survival’ on R (version 4.1.1), a statistical analysis program running on Mac OS X. The animal experiment in this study was approved by the Animal Use Committee at Genome Pharmaceuticals Institute Co., Ltd.

## Results

### First screening of the 1253 PMDA-approved compounds using the silkworm infection model

**Figure 1.**
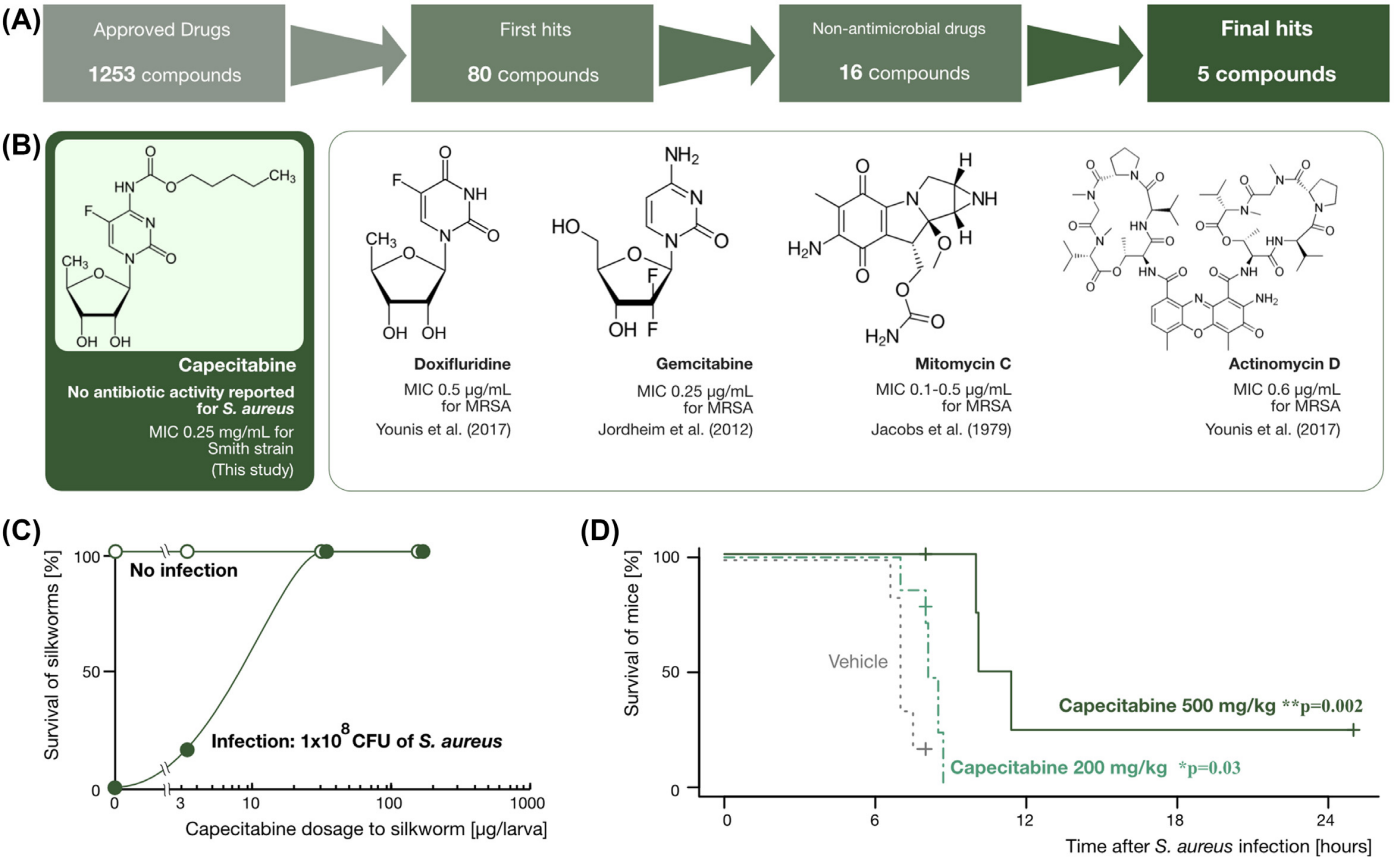
Screening of 1253 PMDA-approved compounds and the therapeutic effects of capecitabine. **(A)** We started the screening with 1253 compounds, obtained 80 candidates in the first screening, 16 compounds among them were nonantibiotics, and eventually obtained five compounds using the silkworm assay system. **(B)** The chemical structures of the five compounds (capecitabine, doxifluridine, gemcitabine, mytomycin C, and actinomycin D) are shown in the panel. Also in the panel, reported information for MIC values are shown with literature reference (but not for capecitabine as there was no information available). In this study, we measured the MIC value of capecitabine for *S. aureus* strains (see Table 1), and it was 0.25 mg/ml for the Smith strain. **(C)** The dose-dependent therapeutic effect of capecitabine in the silkworm model. The *x*-axis represents administered dose of capecitabine (μg/larva), and the *y*-axis represents survival of silkworms (%) at 48 hours after the infection. The closed circles represent capecitabine treated + infection group, and the open circles represent the group treated with capecitabine only (i.e. to assess its toxicity). **(D)** The therapeutic effect of capecitabine in the mouse infection model. Shown in the panel is the survival curves (*n* = 6 (Vehicle), seven (capecitabine 200 mg/kg), and seven (capecitabine 500 mg/kg) with the *x*-axis representing time after infection (hours) and the *y*-axis representing mice survival (%). The differences of survival curves were statistically tested by log-rank test, and the obtained *P*-values are shown in the panel.

To validate the applicability of silkworm infection model to drug repositioning screen, we examined the therapeutic effect of the 1253 PMDA-approved compounds using the silkworm staphylococcal infection model. For this initial screen, compounds that lead to prolonged survival time (i.e. a greater number of silkworms survived at 24 hours compared to the vehicle-injected group) were considered as hit compounds. As a result, 80 out of 1253 compounds were found to exert therapeutic effects on *S. aureus*. Of these, 64 compounds were classified as antimicrobial agents and the remaining 16 compounds were not classified as antimicrobial agents (Fig. 1A, also see Table S1 (Supporting Information) for the list of 80 hits).

### Second screening of the 16 nonantibiotic compounds

To further narrow down the 16 hits, we conducted a re-evaluation of the therapeutic efficacy of the 16 compounds obtained in the first screening. In this second stage of screening, we examined the dose dependence of the therapeutic effect for each compound. Based on this screen, we narrowed down to five compounds that showed reproducible, dose-dependent therapeutic effects in the silkworm model (see Fig. 1A and B). The five compounds were capecitabine, doxifluridine, gemcitabine, mitomycin C, and actinomycin D (see Fig. 1B for their chemical structures). In the literature, growth inhibitory effects on *S. aureus* have been reported for doxifluridine (Younis et al. 2017), gemcitabine (Jordheim et al. 2012), mitomycin C (Jacobs et al. 1979), and actinomycin D (Younis et al. 2017), but not for capecitabine (see Fig. 1B). Therefore, capecitabine was selected for this study, and the therapeutic effects were tested in mice as described in the next section. The dose-dependent therapeutic effect of capecitabine is shown in Fig. 1C (please see Figure S1 (Supporting Information) for the other four compounds). We also measured the MIC values of capecitabine against different *S. aureus* strains as shown in Table 1.

**Table 1.** MIC values of Capecitabine against *S. aureus* strains.

Strain	MIC (µg/ml)
MSSA1	125
MRSA3	250
MRSA4	250
MRSA5	250
MRSA6	250
MRSA8	125
MRSA9	250
MRSA11	250
MRSA12	125
Smith	250
RN4220	63
Newman	125
USA300	250

### Therapeutic effects of capecitabine in mouse models

To validate the therapeutic effect on staphylococcal infection in mammalian animals, we investigated the therapeutic efficacy of capecitabine in mice infected with *S. aureus* using the Smith strain, which is pathogenic to mice. We injected live cells of Smith in the peritoneal cavity of mice, and immediately after that we injected capecitabine at a dose of 200 and 500 mg/kg. As a result, each dose capecitabine had a therapeutic effect on mice, and 500 mg/kg dose had a clearer therapeutic effect (Fig. 1D). The *P*-values for the difference in survival curves between the vehicle group and the capecitabine group in the log-rank test were *P* = .03 for the 200 mg/kg capecitabine group and *P* = .002 for the 500 mg/kg capecitabine group (Fig. 1D).

## Discussion

In this study, we screened 1253 PMDA-approved compounds for their therapeutic effects on infection using the silkworm staphylococcal infection model. We excluded compounds registered as antimicrobials during the screening process and eventually obtained five nonantibiotic compounds: capecitabine, doxifluridine, gemcitabine, mitomycin C, and actinomycin D. Growth inhibitory effects on *S. aureus* has been reported in the literature for four of these compounds [doxifluridine: MIC = 0.5 µg/ml (Younis et al. 2017), gemcitabine: MIC = 0.25 µg/ml (Jordheim et al. 2012), mitomycin C: MIC = 0.1–0.5 µg/ml (Jacobs et al. 1979), and actinomycin D: MIC = 0.6 µg/ml (Younis et al. 2017)], but not for capecitabine. Nevertheless, capecitabine showed a therapeutic effect in prolonging the survival time of mice after intraperitoneal infection with *S. aureus* (200 mg/kg, intraperitoneal administration). Capecitabine has long been used clinically as an anticancer drug, but there is a paucity of literature on its effects on infections. Recently, McLeod et al. (2021) tested 129 compounds (Oncology Drug Set VII) approved as anticancer agents in the United States (FDA-Approved) against *Salmonella enterica* serovar Typhimurium, and found that nine anticancer drugs, including capecitabine, had growth inhibitory effects on *S. enterica* serovar Typhimurium *in vitro*. Among them, capecitabine was found to be effective in inhibiting intravenous *S. aureus* from forming colonies in mice (200 mg/kg, peroral administration). Although there are differences in the pathogen, route of drug administration, and method of determining therapeutic efficacy, our study and the above reports are consistent in suggesting that capecitabine is effective in mammalian models of *S. aureus* infection. Also, in order to understand the mechanism of action *in vivo*, it is important to investigate whether capecitabine inhibits the production of exotoxins and/or the activity of exotoxins.

Based on these findings, it is expected that the risk of *S. aureus* infection is reduced in patients receiving cancer chemotherapy with capecitabine. Patients receiving cancer chemotherapy are generally immunocompromised, and controlling the risk of bacterial infections such as *S. aureus* is an important clinical issue. The use of capecitabine in clinical practice and the risk of *S. aureus* infection may provide important insights for developing more appropriate infection control strategies for individual cases of cancer chemotherapy, which are diverse in terms of the use of capecitabine and differences in patient immunity. Because of the large number of patients receiving anticancer therapy with capecitabine, it would be realistic to conduct an observational study to examine the association between capecitabine use and risk of *S. aureus* infection. Such a study could be designed as a retrospective observational study. The typical dose of capecitabine in clinical practice is 1250 mg/m^2^ twice daily (i.e. 57 mg/kg/day for a patient weighing 70 kg and having a body surface area of 1.6 m^2^), and the maximum plasma concentration achieved for this dose would be 2.5 μg/ml (Reigner et al. 2001). In the silkworm with a typical plasma volume of 0.5 ml, a dose of 3.0 µg/larva (the minimum therapeutic dose as shown in Fig. 1C) should result in a final concentration of 6.0 µg/ml when injected into the hemocoel, assuming that the compound disperses immediately into the plasma. In mice, the expected maximum plasma concentration after a 200 mg/kg oral administration would be 5.8 µg/ml (Onodera et al. 2000), which is comparable, if not adequately low, to the clinically achieved concentration (i.e. 2.5 µg/ml). These values (2.5, 6.0, and 5.8 µg/ml in human, mice, and silkworm, respectively) are more than one order of magnitude lower than the MIC values of capecitabine *in vitro* (see Table 1), which could be a potential shortcoming when expecting clinical significance of capecitabine in the prevention of staphylococcal infection. Nevertheless, such discrepancy between *in vivo* and *in vitro* results may also indicate that the observed therapeutic effects in mice and silkworms are likely due to indirect mechanism (i.e. not direct inhibition of bacterial growth), such as suppression of bacterial exotoxin activity and changes in host immune function. An alternative explanation is that, as with some antimicrobial agents (Hamamoto et al. 2021), host serum factors may had enhanced the antibacterial effect of capecitabine. These points should be investigated further in future studies. In human clinical practice, it is also possible that sustained plasma concentrations from repeated capecitabine administration (as is done in clinical applications) may enhance the therapeutic effect of capecitabine and reduce the risk of staphylococcal infections. Hence, it would be of considerable value to test this hypothesis in a clinical setting.

The use of insect models in drug discovery is important to avoid concerns about cost and animal ethics. In this study, the therapeutic effects of 1253 compounds were screened using live silkworms and narrowed down to one candidate compound (capecitabine). After that, we designed an experiment using a mouse model in order to confirm the therapeutic effects in mammalian system. In recent years, from the viewpoint of animal ethics, screenings that involve large volume of mammalian sacrifice have been regulated. Even if for some reason such restrictions are avoided, it is not realistic to use thousands of mice for the experiment due to the cost and facilities involved*. In vitro* screening can replace animal experiments to a certain extent, but it is difficult to assess pharmacodynamics parameters such as absorption, distribution, metabolism, excretion, and toxicity (ADMET), which are key properties in drug discovery. Nevertheless, in the silkworm model, the ADMET of compounds is often in good agreement with that measured in mammals (Hamamoto et al. 2009, 2019). This means that drug efficacy, including ADMET, can be evaluated at an early stage of drug discovery screening using the silkworm model. This study is the first example of drug repurposing of approved compounds using insect model that confirmed the efficacy of the identified compound in mammalian model. We expect that drug repurposing can be accelerated using other models using the silkworm, such as hyperglycemia model (Matsumoto et al. 2011), and inflammation model (unpublished).

## Supplementary Material

xtac014_Supplemental_File
