# Supplementary material for: Repurposing the PDMA-approved drugs in Japan using an insect model of staphylococcal infection
Source: FEMS Microbes. 2022 Apr 26;3:xtac014. doi: 10.1093/femsmc/xtac014 (PMC10117882; doi:10.1093/femsmc/xtac014)
Supplement: xtac014_Supplemental_File [file xtac014_supplemental_file.docx]

**Supplementary Table S1. Full list of 80 hit compounds obtained in the first screen.**

| Compound Name | Use | Categorized as Antimicrobials? |
| --- | --- | --- |
| Etazolate | Anxiolyic | No |
| Epinastine hydrochloride | Antihistamine | No |
| Alprenolol hydrochloride | antianginal | No |
| Ropinirole Hydrochloride | antiparkinson | No |
| Gemcitabine HCl | Anti-cancer | No |
| Capecitabine | Anti-cancer | No |
| L-Glutamine | Supplement | No |
| Guanfacine hydrochloride | selective α2A receptor agonist | No |
| Fluocinonide | Glucocorticoid Anti-inflammatory Agent | No |
| Crotamiton | Anti-itching Drug | No |
| Doxifluridine | Anti-cancer | No |
| Mitomycin C | Anti-cancer | No |
| Actinomycin D BioChemica | Anti-cancer | No |
| Penbutolol sulfate | non-selective β blocker | No |
| Quinine hydrochloride | For malaria | No |
| Thiamylal sodium | barbiturate derivative | No |
| Biapenem | Carbapenem Antibiotics | Yes |
| Meropenem trihydrate | Carbapenem Antibiotic | Yes |
| Benzylpenicillin Potassium | Penicillin G | Yes |
| Cephalexin | β-lactam Antibiotic | Yes |
| Doripenem Hydrate | Carbapenem Antibiotic | Yes |
| Cefaclor | Second-generation Cephalosporin | Yes |
| Cloxacillin Sodium | Penicillin Antibiotic | Yes |
| Bacampicillin Hydrochloride | Prodrug of Ampicillin | Yes |
| Sarafloxacin HCl | Quinolone Antibiotic | Yes |
| Demethyltetracycline Hydrochloride | Tetracycline Antibiotic | Yes |
| Gentamycin sulfate | Aminoglycoside Antibiotic | Yes |
| Vancomycin Hydrochloride | Glycopeptide Antibiotic | Yes |
| Arbekacin Sulphate | Aminoglycoside Antibiotic | Yes |
| Amoxicillin | β-lactam Antibiotic | Yes |
| Aspoxicillin | β-lactam Antibiotic | Yes |
| Netilmicin sulate | Aminoglycoside Antibiotic | Yes |
| Moixifloxacin Hydrochloride | Fluoroquinolone Antibiotic | Yes |
| Daptomycin | Lipopeptide Antiiotic | Yes |
| Teicoplanin | Glycopeptide Antibiotic | Yes |
| Doxycycline Hyclate | Tetracycline Antibiotic | Yes |
| Fradiomycin Sulfate | Aminoglycoside Antibiotic | Yes |
| Cefpirome sulfate | Cephalosporin Antibiotic | Yes |
| Pazufloxacin mesilate | Fluoroquinolone Antibiotic | Yes |
| Sulfadimethoxine | Sulfonamine Antibiotic | Yes |
| Cefalotin sodium | Cephalosporin Antibiotic | Yes |
| Ampicillin | Broad-spectrum beta-lactam Antibioic | Yes |
| Clindamycin | Lincosamide Antibiotic | Yes |
| Clinafloxacin hydrochloride | Fluoroquinolone Antibiotic | Yes |
| Levofloxacin | Fluoroquinolone Antibiotic | Yes |
| Sulbenicillin sodium | Penicillin Antibiotic | Yes |
| Novobiocin sodium | Aminocoumarin Antibiotic | Yes |
| Amoxicillin | aminopenicillin Antibiotic | Yes |
| Cefazolin sodium salt | first-generation cephalosporin Antibiotic | Yes |
| Cefotaxime sodium | Beta-lactam Antibiotic | Yes |
| Oxytetracycline hydrochloride | Tetracycline Antibiotic | Yes |
| Calcium mupirocin dihydrate | Topical Antibiotic | Yes |
| Cefdinir | Third-generation Cephalosporin Antibiotic | Yes |
| Cefcapene pivoxil hydrochloride | Cephalosporin Antibiotic | Yes |
| Gatifloxacin | Fluoroquinolone Antibiotic | Yes |
| Rifampicin | Antibiotic | Yes |
| Ceftriaxone sodium | Third-generation Cephalosporin Antibiotic | Yes |
| Cefditoren pivoxil | Third-generation Cephalosporin Antibiotic | Yes |
| Cefsulodin sodium salt hydrate | Third-generation Cephalosporin Antibiotic | Yes |
| Cefteram pivoxil | Third-generation Cephalosporin Antibiotic | Yes |
| Rifabutin | For Tuberculosis | Yes |
| Cefotiam Hydrochloride | Second-generation Cephalosporin | Yes |
| Faropenem sodium hydrate | Beta-lactam Antibiotic | Yes |
| Cefminox Sodium Heptahydrate | Second-generation Cephalosporin | Yes |
| Cefpodoxime Proxetil | Third-generation Cephalosporin Antibiotic | Yes |
| Cefepime Dihydrochloride Monohydrate | Fourth-generation Cephalosporin Antibiotic | Yes |
| Cefotiam Hexetil Hydrochloride | Second-generation Cephalosporin | Yes |
| Cefmenoxime Hydrochloride | Third-generation Cephalosporin Antibiotic | Yes |
| Cefuroxime Axetil | Second-generation Cephalosporin | Yes |
| Roxithromycin | Macrolide antibiotic | Yes |
| Tetracycline hydrochloride | Tetracycline Antibiotic | Yes |
| Tylosin tartrate | Macrolide antibiotic | Yes |
| Gentamycin sulfate | Aminoglycoside Antibiotic | Yes |
| Troleandomycin | Macrolide antibiotic | Yes |
| Ampicillin trihydrate | Beta-lactam Antibiotic | Yes |
| Erythromycin | Macrolide antibiotic | Yes |
| Rifamycin SV | For Tuberculosis | Yes |
| Thiamphenicol glycinate | Analogue of chloramphenicol | Yes |
| Piperacillin | Ureidopenicillin class beta-lactam antibiotic | Yes |
| Cefixime | Third-generation Cephalosporin Antibiotic | Yes |


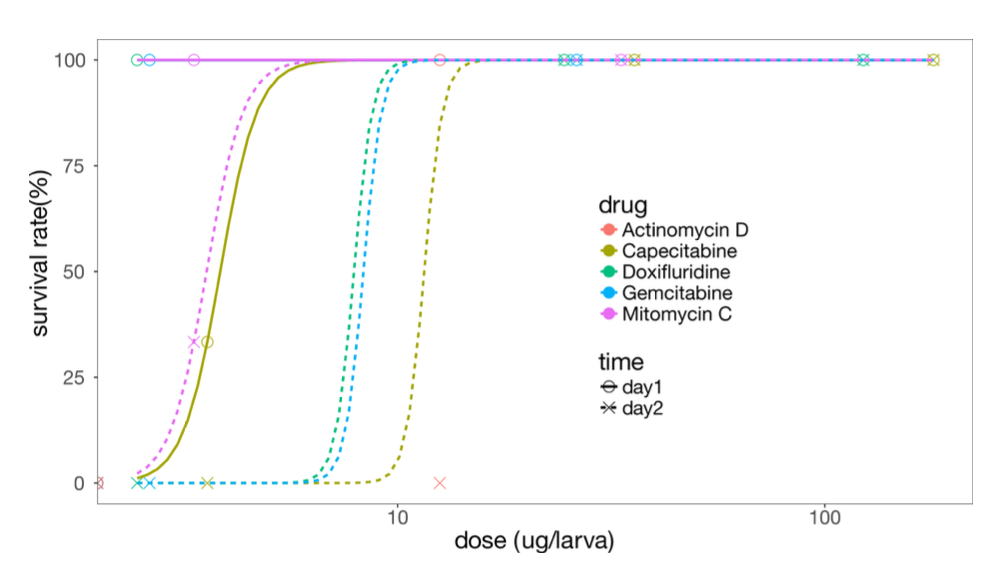


**Supplementary Figure S1. Therapeutic Effects of five anticancer drugs on staphylococcal infection model using the silkworm (*Bombyx mori*).** Silkworms were injected with live staphylococcal cells followed by each cancer drug as shown in the panel. The horizontal axis represents the dose of each drug administered to the silkworm (µg/larva), and the vertical axis represents the survival of silkworms (%) 24 and 48 hours after the infection.
